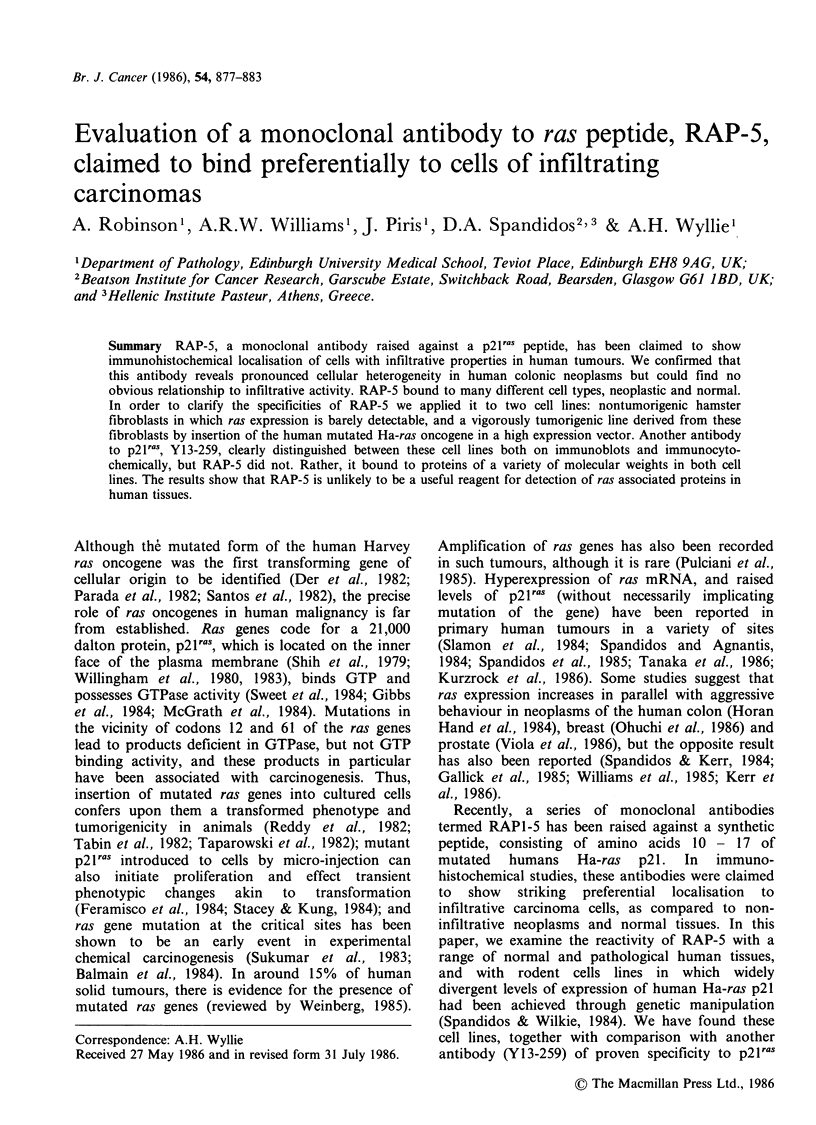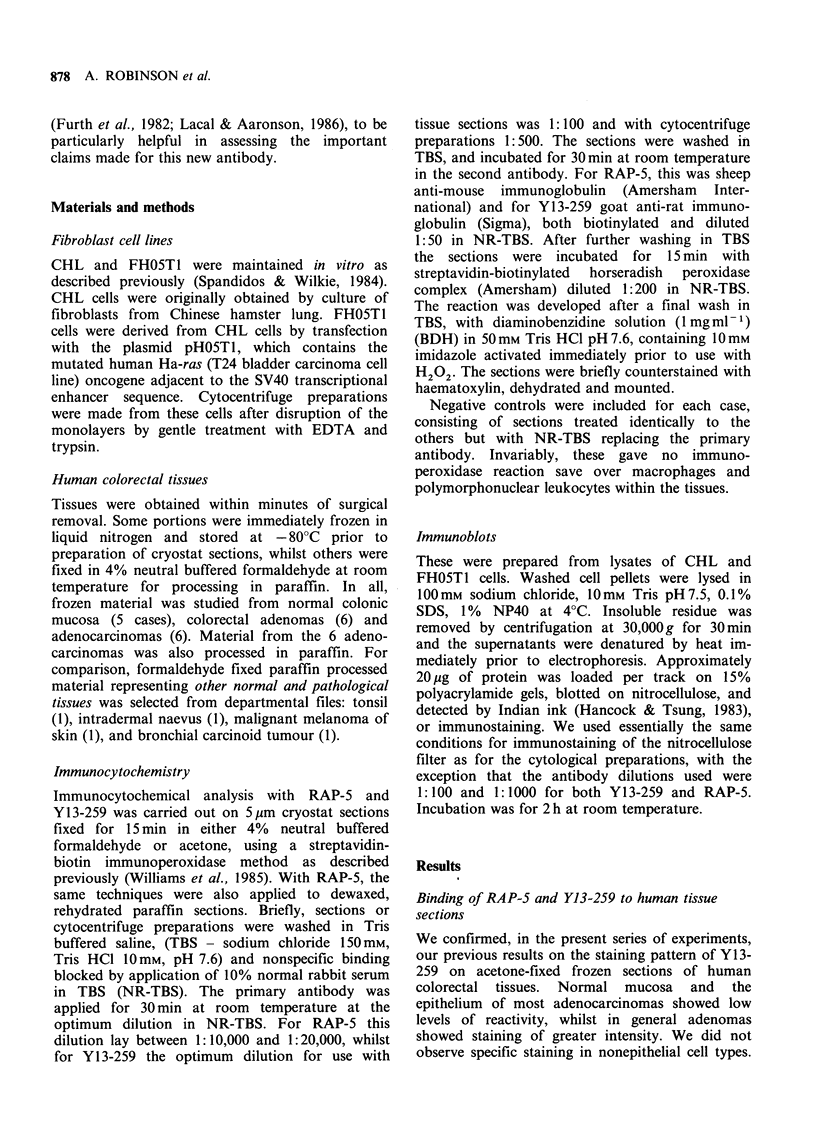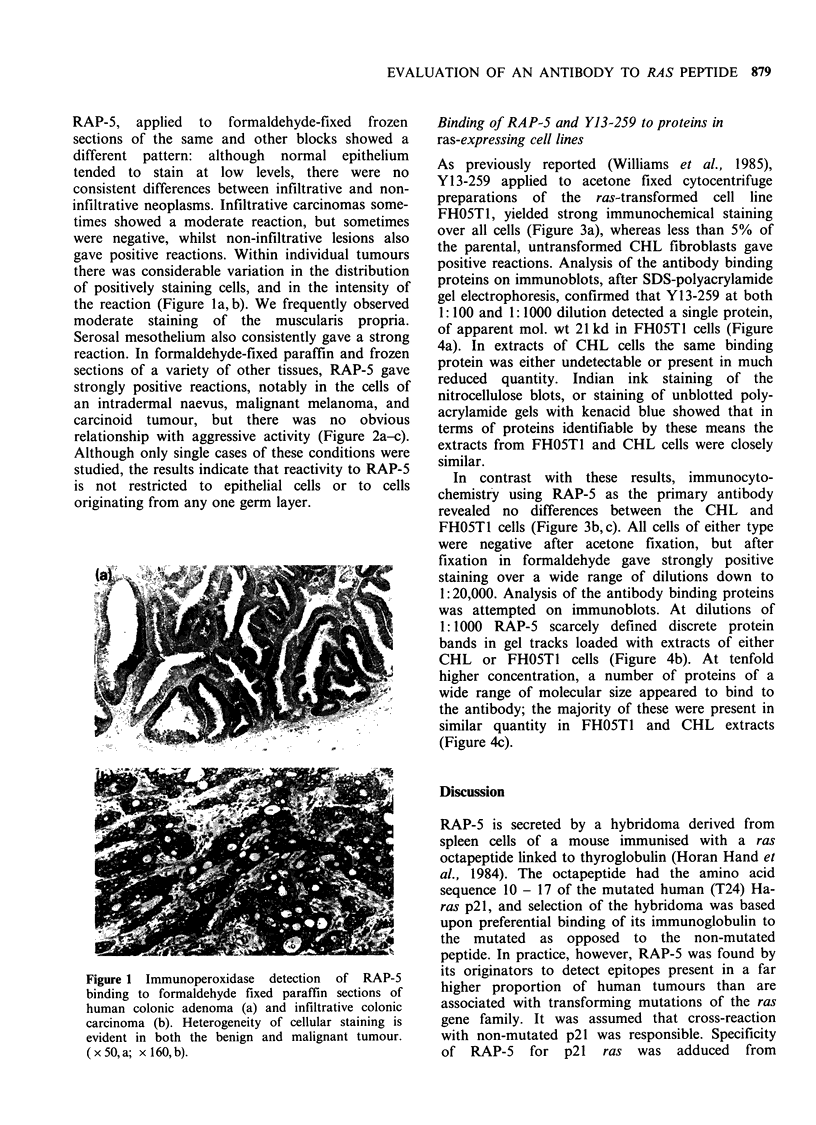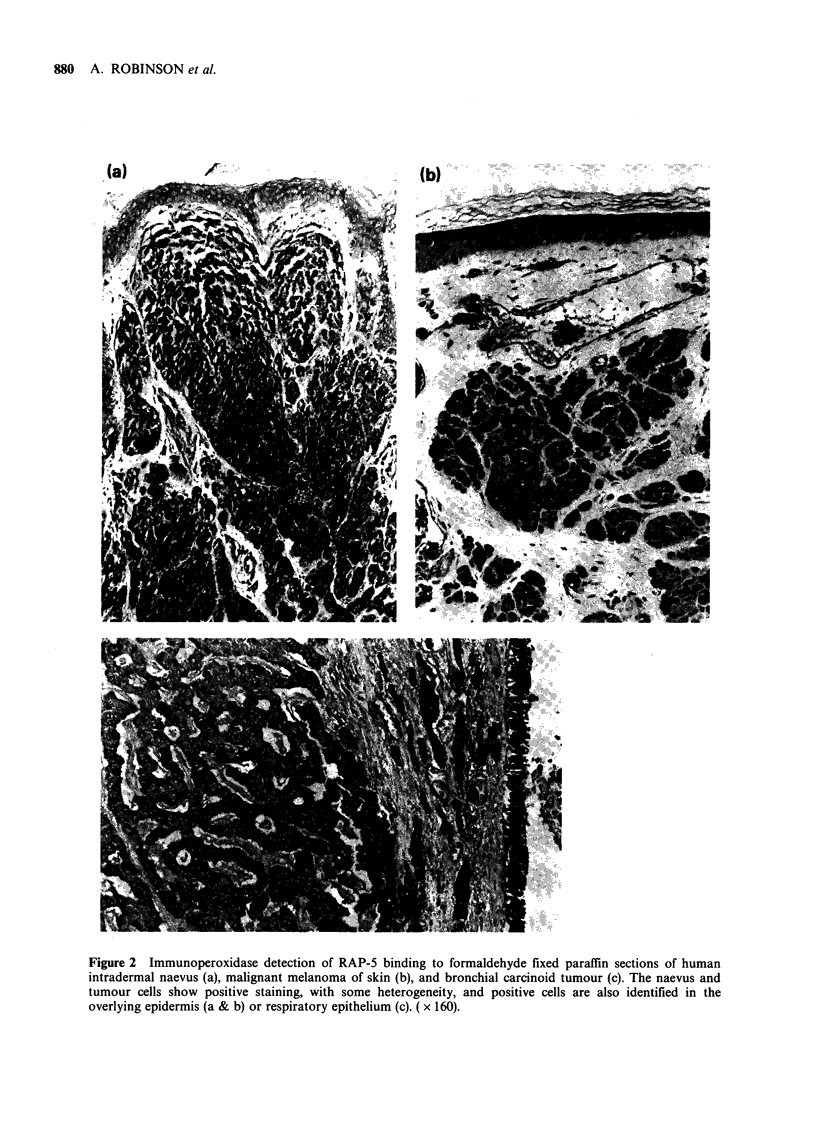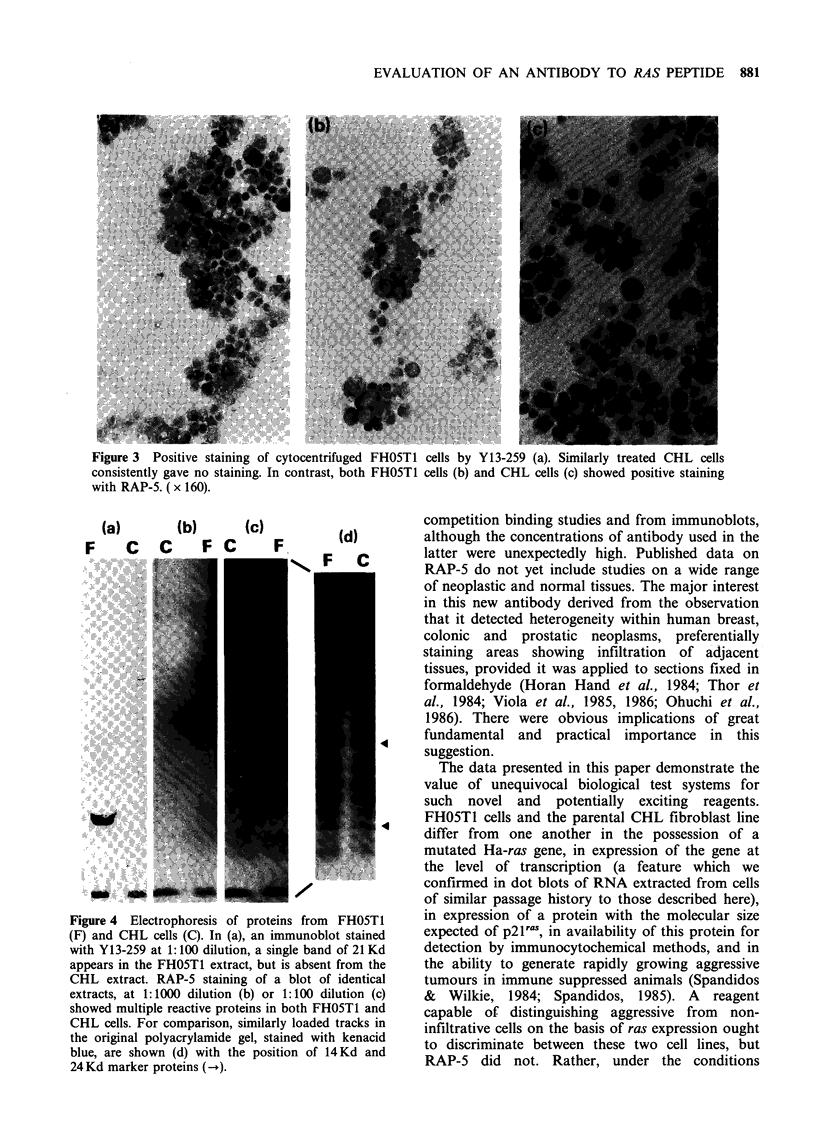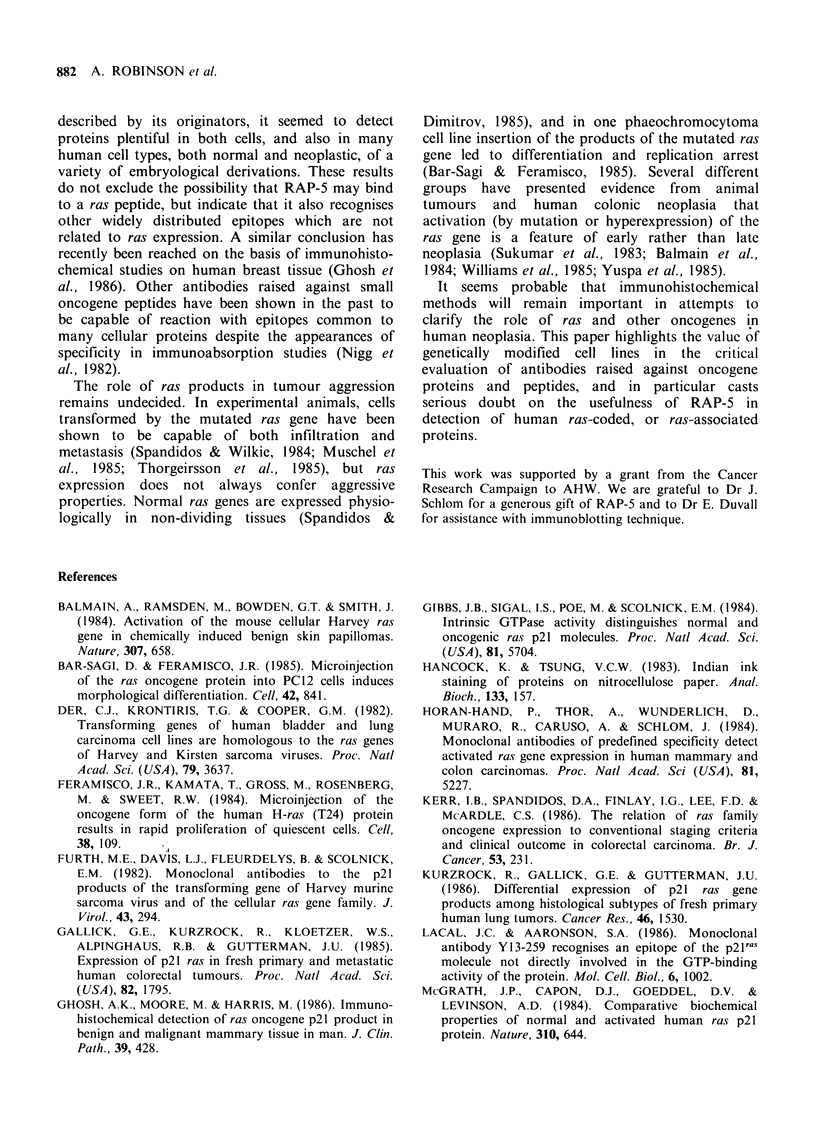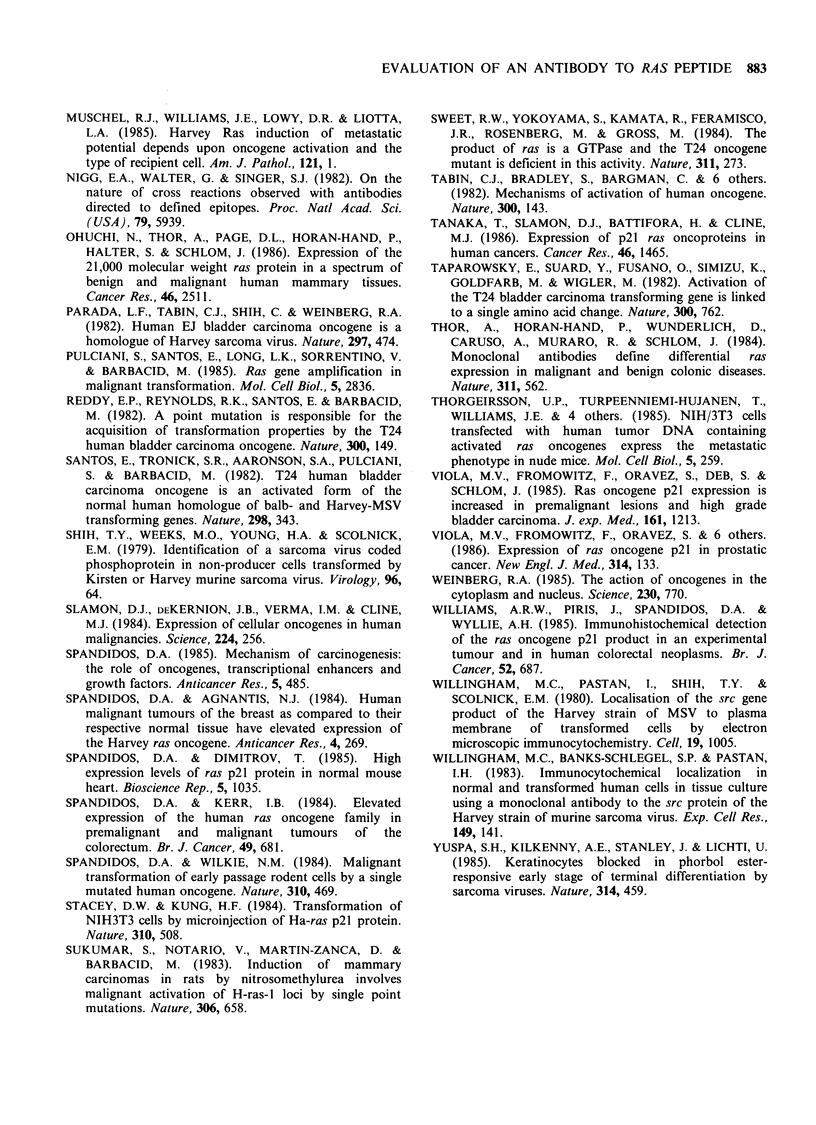# Evaluation of a monoclonal antibody to ras peptide, RAP-5, claimed to bind preferentially to cells of infiltrating carcinomas.

**DOI:** 10.1038/bjc.1986.256

**Published:** 1986-12

**Authors:** A. Robinson, A. R. Williams, J. Piris, D. A. Spandidos, A. H. Wyllie

## Abstract

**Images:**


Br. J. Cancer (1986), 54, 877–883

### Evaluation of a monoclonal antibody to *ras* peptide, RAP-5, claimed to bind preferentially to cells of infiltrating carcinomas

A. Robinson[1], A.R.W. Williams[1], J. Piris[1], D.A. Spandidos[2,3] & A.H. Wyllie[1]

[1]*Department of Pathology, Edinburgh University Medical School, Teviot Place, Edinburgh EH8 9AG, UK;* [2]*Beatson Institute for Cancer Research, Garscube Estate, Switchback Road, Bearsden, Glasgow G61 1BD, UK;* and [3]*Hellenic Institute Pasteur, Athens, Greece.*

**Summary** RAP-5, a monoclonal antibody raised against a p21$^{ras}$ peptide, has been claimed to show immunohistochemical localisation of cells with infiltrative properties in human tumours. We confirmed that this antibody reveals pronounced cellular heterogeneity in human colonic neoplasms but could find no obvious relationship to infiltrative activity. RAP-5 bound to many different cell types, neoplastic and normal. In order to clarify the specificities of RAP-5 we applied it to two cell lines: nontumorigenic hamster fibroblasts in which *ras* expression is barely detectable, and a vigorously tumorigenic line derived from these fibroblasts by insertion of the human mutated Ha-*ras* oncogene in a high expression vector. Another antibody to p21$^{ras}$, Y13-259, clearly distinguished between these cell lines both on immunoblots and immunocytochemically, but RAP-5 did not. Rather, it bound to proteins of a variety of molecular weights in both cell lines. The results show that RAP-5 is unlikely to be a useful reagent for detection of *ras* associated proteins in human tissues.

Although the mutated form of the human Harvey *ras* oncogene was the first transforming gene of cellular origin to be identified (Der *et al.*, 1982; Parada *et al.*, 1982; Santos *et al.*, 1982), the precise role of *ras* oncogenes in human malignancy is far from established. *Ras* genes code for a 21,000 dalton protein, p21$^{ras}$, which is located on the inner face of the plasma membrane (Shih *et al.*, 1979; Willingham *et al.*, 1980, 1983), binds GTP and possesses GTPase activity (Sweet *et al.*, 1984; Gibbs *et al.*, 1984; McGrath *et al.*, 1984). Mutations in the vicinity of codons 12 and 61 of the *ras* genes lead to products deficient in GTPase, but not GTP binding activity, and these products in particular have been associated with carcinogenesis. Thus, insertion of mutated *ras* genes into cultured cells confers upon them a transformed phenotype and tumorigenicity in animals (Reddy *et al.*, 1982; Tabin *et al.*, 1982; Taparowski *et al.*, 1982); mutant p21$^{ras}$ introduced to cells by micro-injection can also initiate proliferation and effect transient phenotypic changes akin to transformation (Feramisco *et al.*, 1984; Stacey & Kung, 1984); and *ras* gene mutation at the critical sites has been shown to be an early event in experimental chemical carcinogenesis (Sukumar *et al.*, 1983; Balmain *et al.*, 1984). In around 15% of human solid tumours, there is evidence for the presence of mutated *ras* genes (reviewed by Weinberg, 1985).

Correspondence: A.H. Wyllie
Received 27 May 1986 and in revised form 31 July 1986.

Amplification of *ras* genes has also been recorded in such tumours, although it is rare (Pulciani *et al.*, 1985). Hyperexpression of *ras* mRNA, and raised levels of p21$^{ras}$ (without necessarily implicating mutation of the gene) have been reported in primary human tumours in a variety of sites (Slamon *et al.*, 1984; Spandidos and Agnantis, 1984; Spandidos *et al.*, 1985; Tanaka *et al.*, 1986; Kurzrock *et al.*, 1986). Some studies suggest that *ras* expression increases in parallel with aggressive behaviour in neoplasms of the human colon (Horan Hand *et al.*, 1984), breast (Ohuchi *et al.*, 1986) and prostate (Viola *et al.*, 1986), but the opposite result has also been reported (Spandidos & Kerr, 1984; Gallick *et al.*, 1985; Williams *et al.*, 1985; Kerr *et al.*, 1986).

Recently, a series of monoclonal antibodies termed RAP1-5 has been raised against a synthetic peptide, consisting of amino acids 10 – 17 of mutated humans Ha-*ras* p21. In immunohistochemical studies, these antibodies were claimed to show striking preferential localisation to infiltrative carcinoma cells, as compared to non-infiltrative neoplasms and normal tissues. In this paper, we examine the reactivity of RAP-5 with a range of normal and pathological human tissues, and with rodent cells lines in which widely divergent levels of expression of human Ha-*ras* p21 had been achieved through genetic manipulation (Spandidos & Wilkie, 1984). We have found these cell lines, together with comparison with another antibody (Y13-259) of proven specificity to p21$^{ras}$

© The Macmillan Press Ltd., 1986

(Furth *et al.*, 1982; Lacal & Aaronson, 1986), to be particularly helpful in assessing the important claims made for this new antibody.

### Materials and methods

### *Fibroblast cell lines*

CHL and FH05T1 were maintained *in vitro* as described previously (Spandidos & Wilkie, 1984). CHL cells were originally obtained by culture of fibroblasts from Chinese hamster lung. FH05T1 cells were derived from CHL cells by transfection with the plasmid pH05T1, which contains the mutated human Ha-*ras* (T24 bladder carcinoma cell line) oncogene adjacent to the SV40 transcriptional enhancer sequence. Cytocentrifuge preparations were made from these cells after disruption of the monolayers by gentle treatment with EDTA and trypsin.

### *Human colorectal tissues*

Tissues were obtained within minutes of surgical removal. Some portions were immediately frozen in liquid nitrogen and stored at −80°C prior to preparation of cryostat sections, whilst others were fixed in 4% neutral buffered formaldehyde at room temperature for processing in paraffin. In all, frozen material was studied from normal colonic mucosa (5 cases), colorectal adenomas (6) and adenocarcinomas (6). Material from the 6 adenocarcinomas was also processed in paraffin. For comparison, formaldehyde fixed paraffin processed material representing *other normal and pathological tissues* was selected from departmental files: tonsil (1), intradermal naevus (1), malignant melanoma of skin (1), and bronchial carcinoid tumour (1).

### *Immunocytochemistry*

Immunocytochemical analysis with RAP-5 and Y13-259 was carried out on 5 μm cryostat sections fixed for 15 min in either 4% neutral buffered formaldehyde or acetone, using a streptavidin-biotin immunoperoxidase method as described previously (Williams *et al.*, 1985). With RAP-5, the same techniques were also applied to dewaxed, rehydrated paraffin sections. Briefly, sections or cytocentrifuge preparations were washed in Tris buffered saline, (TBS – sodium chloride 150 mM, Tris HCl 10 mM, pH 7.6) and nonspecific binding blocked by application of 10% normal rabbit serum in TBS (NR-TBS). The primary antibody was applied for 30 min at room temperature at the optimum dilution in NR-TBS. For RAP-5 this dilution lay between 1:10,000 and 1:20,000, whilst for Y13-259 the optimum dilution for use with tissue sections was 1:100 and with cytocentrifuge preparations 1:500. The sections were washed in TBS, and incubated for 30 min at room temperature in the second antibody. For RAP-5, this was sheep anti-mouse immunoglobulin (Amersham International) and for Y13-259 goat anti-rat immunoglobulin (Sigma), both biotinylated and diluted 1:50 in NR-TBS. After further washing in TBS the sections were incubated for 15 min with streptavidin-biotinylated horseradish peroxidase complex (Amersham) diluted 1:200 in NR-TBS. The reaction was developed after a final wash in TBS, with diaminobenzidine solution (1 mg ml$^{-1}$) (BDH) in 50 mM Tris HCl pH 7.6, containing 10 mM imidazole activated immediately prior to use with $H_2O_2$. The sections were briefly counterstained with haematoxylin, dehydrated and mounted.

Negative controls were included for each case, consisting of sections treated identically to the others but with NR-TBS replacing the primary antibody. Invariably, these gave no immunoperoxidase reaction save over macrophages and polymorphonuclear leukocytes within the tissues.

### *Immunoblots*

These were prepared from lysates of CHL and FH05T1 cells. Washed cell pellets were lysed in 100 mM sodium chloride, 10 mM Tris pH 7.5, 0.1% SDS, 1% NP40 at 4°C. Insoluble residue was removed by centrifugation at 30,000 *g* for 30 min and the supernatants were denatured by heat immediately prior to electrophoresis. Approximately 20 μg of protein was loaded per track on 15% polyacrylamide gels, blotted on nitrocellulose, and detected by Indian ink (Hancock & Tsung, 1983), or immunostaining. We used essentially the same conditions for immunostaining of the nitrocellulose filter as for the cytological preparations, with the exception that the antibody dilutions used were 1:100 and 1:1000 for both Y13-259 and RAP-5. Incubation was for 2 h at room temperature.

### Results

### *Binding of RAP-5 and Y13-259 to human tissue sections*

We confirmed, in the present series of experiments, our previous results on the staining pattern of Y13-259 on acetone-fixed frozen sections of human colorectal tissues. Normal mucosa and the epithelium of most adenocarcinomas showed low levels of reactivity, whilst in general adenomas showed staining of greater intensity. We did not observe specific staining in nonepithelial cell types.

RAP-5, applied to formaldehyde-fixed frozen sections of the same and other blocks showed a different pattern: although normal epithelium tended to stain at low levels, there were no consistent differences between infiltrative and non-infiltrative neoplasms. Infiltrative carcinomas sometimes showed a moderate reaction, but sometimes were negative, whilst non-infiltrative lesions also gave positive reactions. Within individual tumours there was considerable variation in the distribution of positively staining cells, and in the intensity of the reaction (Figure 1a, b). We frequently observed moderate staining of the muscularis propria. Serosal mesothelium also consistently gave a strong reaction. In formaldehyde-fixed paraffin and frozen sections of a variety of other tissues, RAP-5 gave strongly positive reactions, notably in the cells of an intradermal naevus, malignant melanoma, and carcinoid tumour, but there was no obvious relationship with aggressive activity (Figure 2a–c). Although only single cases of these conditions were studied, the results indicate that reactivity to RAP-5 is not restricted to epithelial cells or to cells originating from any one germ layer.

**Figure 1** Immunoperoxidase detection of RAP-5 binding to formaldehyde fixed paraffin sections of human colonic adenoma (a) and infiltrative colonic carcinoma (b). Heterogeneity of cellular staining is evident in both the benign and malignant tumour. (×50, a; ×160, b).

*Binding of RAP-5 and Y13-259 to proteins in* ras*-expressing cell lines*

As previously reported (Williams *et al.*, 1985), Y13-259 applied to acetone fixed cytocentrifuge preparations of the *ras*-transformed cell line FH05T1, yielded strong immunochemical staining over all cells (Figure 3a), whereas less than 5% of the parental, untransformed CHL fibroblasts gave positive reactions. Analysis of the antibody binding proteins on immunoblots, after SDS-polyacrylamide gel electrophoresis, confirmed that Y13-259 at both 1:100 and 1:1000 dilution detected a single protein, of apparent mol. wt 21 kd in FH05T1 cells (Figure 4a). In extracts of CHL cells the same binding protein was either undetectable or present in much reduced quantity. Indian ink staining of the nitrocellulose blots, or staining of unblotted polyacrylamide gels with kenacid blue showed that in terms of proteins identifiable by these means the extracts from FH05T1 and CHL cells were closely similar.

In contrast with these results, immunocytochemistry using RAP-5 as the primary antibody revealed no differences between the CHL and FH05T1 cells (Figure 3b, c). All cells of either type were negative after acetone fixation, but after fixation in formaldehyde gave strongly positive staining over a wide range of dilutions down to 1:20,000. Analysis of the antibody binding proteins was attempted on immunoblots. At dilutions of 1:1000 RAP-5 scarcely defined discrete protein bands in gel tracks loaded with extracts of either CHL or FH05T1 cells (Figure 4b). At tenfold higher concentration, a number of proteins of a wide range of molecular size appeared to bind to the antibody; the majority of these were present in similar quantity in FH05T1 and CHL extracts (Figure 4c).

### Discussion

RAP-5 is secreted by a hybridoma derived from spleen cells of a mouse immunised with a *ras* octapeptide linked to thyroglobulin (Horan Hand *et al.*, 1984). The octapeptide had the amino acid sequence 10 – 17 of the mutated human (T24) Ha-*ras* p21, and selection of the hybridoma was based upon preferential binding of its immunoglobulin to the mutated as opposed to the non-mutated peptide. In practice, however, RAP-5 was found by its originators to detect epitopes present in a far higher proportion of human tumours than are associated with transforming mutations of the *ras* gene family. It was assumed that cross-reaction with non-mutated p21 was responsible. Specificity of RAP-5 for p21 *ras* was adduced from

**Figure 2** Immunoperoxidase detection of RAP-5 binding to formaldehyde fixed paraffin sections of human intradermal naevus (a), malignant melanoma of skin (b), and bronchial carcinoid tumour (c). The naevus and tumour cells show positive staining, with some heterogeneity, and positive cells are also identified in the overlying epidermis (a & b) or respiratory epithelium (c). ( × 160).

**Figure 3** Positive staining of cytocentrifuged FH05T1 cells by Y13-259 (a). Similarly treated CHL cells consistently gave no staining. In contrast, both FH05T1 cells (b) and CHL cells (c) showed positive staining with RAP-5. ( × 160).

**Figure 4** Electrophoresis of proteins from FH05T1 (F) and CHL cells (C). In (a), an immunoblot stained with Y13-259 at 1:100 dilution, a single band of 21 Kd appears in the FH05T1 extract, but is absent from the CHL extract. RAP-5 staining of a blot of identical extracts, at 1:1000 dilution (b) or 1:100 dilution (c) showed multiple reactive proteins in both FH05T1 and CHL cells. For comparison, similarly loaded tracks in the original polyacrylamide gel, stained with kenacid blue, are shown (d) with the position of 14 Kd and 24 Kd marker proteins (→).

competition binding studies and from immunoblots, although the concentrations of antibody used in the latter were unexpectedly high. Published data on RAP-5 do not yet include studies on a wide range of neoplastic and normal tissues. The major interest in this new antibody derived from the observation that it detected heterogeneity within human breast, colonic and prostatic neoplasms, preferentially staining areas showing infiltration of adjacent tissues, provided it was applied to sections fixed in formaldehyde (Horan Hand *et al.*, 1984; Thor *et al.*, 1984; Viola *et al.*, 1985, 1986; Ohuchi *et al.*, 1986). There were obvious implications of great fundamental and practical importance in this suggestion.

The data presented in this paper demonstrate the value of unequivocal biological test systems for such novel and potentially exciting reagents. FH05T1 cells and the parental CHL fibroblast line differ from one another in the possession of a mutated Ha-*ras* gene, in expression of the gene at the level of transcription (a feature which we confirmed in dot blots of RNA extracted from cells of similar passage history to those described here), in expression of a protein with the molecular size expected of $p21^{ras}$, in availability of this protein for detection by immunocytochemical methods, and in the ability to generate rapidly growing aggressive tumours in immune suppressed animals (Spandidos & Wilkie, 1984; Spandidos, 1985). A reagent capable of distinguishing aggressive from non-infiltrative cells on the basis of *ras* expression ought to discriminate between these two cell lines, but RAP-5 did not. Rather, under the conditions described by its originators, it seemed to detect proteins plentiful in both cells, and also in many human cell types, both normal and neoplastic, of a variety of embryological derivations. These results do not exclude the possibility that RAP-5 may bind to a *ras* peptide, but indicate that it also recognises other widely distributed epitopes which are not related to *ras* expression. A similar conclusion has recently been reached on the basis of immunohistochemical studies on human breast tissue (Ghosh *et al.*, 1986). Other antibodies raised against small oncogene peptides have been shown in the past to be capable of reaction with epitopes common to many cellular proteins despite the appearances of specificity in immunoabsorption studies (Nigg *et al.*, 1982).

The role of *ras* products in tumour aggression remains undecided. In experimental animals, cells transformed by the mutated *ras* gene have been shown to be capable of both infiltration and metastasis (Spandidos & Wilkie, 1984; Muschel *et al.*, 1985; Thorgeirsson *et al.*, 1985), but *ras* expression does not always confer aggressive properties. Normal *ras* genes are expressed physiologically in non-dividing tissues (Spandidos & Dimitrov, 1985), and in one phaeochromocytoma cell line insertion of the products of the mutated *ras* gene led to differentiation and replication arrest (Bar-Sagi & Feramisco, 1985). Several different groups have presented evidence from animal tumours and human colonic neoplasia that activation (by mutation or hyperexpression) of the *ras* gene is a feature of early rather than late neoplasia (Sukumar *et al.*, 1983; Balmain *et al.*, 1984; Williams *et al.*, 1985; Yuspa *et al.*, 1985).

It seems probable that immunohistochemical methods will remain important in attempts to clarify the role of *ras* and other oncogenes in human neoplasia. This paper highlights the value of genetically modified cell lines in the critical evaluation of antibodies raised against oncogene proteins and peptides, and in particular casts serious doubt on the usefulness of RAP-5 in detection of human *ras*-coded, or *ras*-associated proteins.

This work was supported by a grant from the Cancer Research Campaign to AHW. We are grateful to Dr J. Schlom for a generous gift of RAP-5 and to Dr E. Duvall for assistance with immunoblotting technique.

### References

BALMAIN, A., RAMSDEN, M., BOWDEN, G.T. & SMITH, J. (1984). Activation of the mouse cellular Harvey *ras* gene in chemically induced benign skin papillomas. *Nature*, **307**, 658.

BAR-SAGI, D. & FERAMISCO, J.R. (1985). Microinjection of the *ras* oncogene protein into PC12 cells induces morphological differentiation. *Cell*, **42**, 841.

DER, C.J., KRONTIRIS, T.G. & COOPER, G.M. (1982). Transforming genes of human bladder and lung carcinoma cell lines are homologous to the *ras* genes of Harvey and Kirsten sarcoma viruses. *Proc. Natl Acad. Sci.* (*USA*), **79**, 3637.

FERAMISCO, J.R., KAMATA, T., GROSS, M., ROSENBERG, M. & SWEET, R.W. (1984). Microinjection of the oncogene form of the human H-*ras* (T24) protein results in rapid proliferation of quiescent cells. *Cell*, **38**, 109.

FURTH, M.E., DAVIS, L.J., FLEURDELYS, B. & SCOLNICK, E.M. (1982). Monoclonal antibodies to the p21 products of the transforming gene of Harvey murine sarcoma virus and of the cellular *ras* gene family. *J. Virol.*, **43**, 294.

GALLICK, G.E., KURZROCK, R., KLOETZER, W.S., ALPINGHAUS, R.B. & GUTTERMAN, J.U. (1985). Expression of p21 *ras* in fresh primary and metastatic human colorectal tumours. *Proc. Natl Acad. Sci.* (*USA*), **82**, 1795.

GHOSH, A.K., MOORE, M. & HARRIS, M. (1986). Immunohistochemical detection of *ras* oncogene p21 product in benign and malignant mammary tissue in man. *J. Clin. Path.*, **39**, 428.

GIBBS, J.B., SIGAL, I.S., POE, M. & SCOLNICK, E.M. (1984). Intrinsic GTPase activity distinguishes normal and oncogenic *ras* p21 molecules. *Proc. Natl Acad. Sci.* (*USA*), **81**, 5704.

HANCOCK, K. & TSUNG, V.C.W. (1983). Indian ink staining of proteins on nitrocellulose paper. *Anal. Bioch.*, **133**, 157.

HORAN-HAND, P., THOR, A., WUNDERLICH, D., MURARO, R., CARUSO, A. & SCHLOM, J. (1984). Monoclonal antibodies of predefined specificity detect activated *ras* gene expression in human mammary and colon carcinomas. *Proc. Natl Acad. Sci* (*USA*), **81**, 5227.

KERR, I.B., SPANDIDOS, D.A., FINLAY, I.G., LEE, F.D. & McARDLE, C.S. (1986). The relation of *ras* family oncogene expression to conventional staging criteria and clinical outcome in colorectal carcinoma. *Br. J. Cancer*, **53**, 231.

KURZROCK, R., GALLICK, G.E. & GUTTERMAN, J.U. (1986). Differential expression of p21 *ras* gene products among histological subtypes of fresh primary human lung tumors. *Cancer Res.*, **46**, 1530.

LACAL, J.C. & AARONSON, S.A. (1986). Monoclonal antibody Y13-259 recognises an epitope of the $p21^{ras}$ molecule not directly involved in the GTP-binding activity of the protein. *Mol. Cell. Biol.*, **6**, 1002.

McGRATH, J.P., CAPON, D.J., GOEDDEL, D.V. & LEVINSON, A.D. (1984). Comparative biochemical properties of normal and activated human *ras* p21 protein. *Nature*, **310**, 644.

MUSCHEL, R.J., WILLIAMS, J.E., LOWY, D.R. & LIOTTA, L.A. (1985). Harvey Ras induction of metastatic potential depends upon oncogene activation and the type of recipient cell. *Am. J. Pathol.*, **121**, 1.

NIGG, E.A., WALTER, G. & SINGER, S.J. (1982). On the nature of cross reactions observed with antibodies directed to defined epitopes. *Proc. Natl Acad. Sci. (USA)*, **79**, 5939.

OHUCHI, N., THOR, A., PAGE, D.L., HORAN-HAND, P., HALTER, S. & SCHLOM, J. (1986). Expression of the 21,000 molecular weight *ras* protein in a spectrum of benign and malignant human mammary tissues. *Cancer Res.*, **46**, 2511.

PARADA, L.F., TABIN, C.J., SHIH, C. & WEINBERG, R.A. (1982). Human EJ bladder carcinoma oncogene is a homologue of Harvey sarcoma virus. *Nature*, **297**, 474.

PULCIANI, S., SANTOS, E., LONG, L.K., SORRENTINO, V. & BARBACID, M. (1985). *Ras* gene amplification in malignant transformation. *Mol. Cell Biol.*, **5**, 2836.

REDDY, E.P., REYNOLDS, R.K., SANTOS, E. & BARBACID, M. (1982). A point mutation is responsible for the acquisition of transformation properties by the T24 human bladder carcinoma oncogene. *Nature*, **300**, 149.

SANTOS, E., TRONICK, S.R., AARONSON, S.A., PULCIANI, S. & BARBACID, M. (1982). T24 human bladder carcinoma oncogene is an activated form of the normal human homologue of balb- and Harvey-MSV transforming genes. *Nature*, **298**, 343.

SHIH, T.Y., WEEKS, M.O., YOUNG, H.A. & SCOLNICK, E.M. (1979). Identification of a sarcoma virus coded phosphoprotein in non-producer cells transformed by Kirsten or Harvey murine sarcoma virus. *Virology*, **96**, 64.

SLAMON, D.J., DEKERNION, J.B., VERMA, I.M. & CLINE, M.J. (1984). Expression of cellular oncogenes in human malignancies. *Science*, **224**, 256.

SPANDIDOS, D.A. (1985). Mechanism of carcinogenesis: the role of oncogenes, transcriptional enhancers and growth factors. *Anticancer Res.*, **5**, 485.

SPANDIDOS, D.A. & AGNANTIS, N.J. (1984). Human malignant tumours of the breast as compared to their respective normal tissue have elevated expression of the Harvey *ras* oncogene. *Anticancer Res.*, **4**, 269.

SPANDIDOS, D.A. & DIMITROV, T. (1985). High expression levels of *ras* p21 protein in normal mouse heart. *Bioscience Rep.*, **5**, 1035.

SPANDIDOS, D.A. & KERR, I.B. (1984). Elevated expression of the human *ras* oncogene family in premalignant and malignant tumours of the colorectum. *Br. J. Cancer*, **49**, 681.

SPANDIDOS, D.A. & WILKIE, N.M. (1984). Malignant transformation of early passage rodent cells by a single mutated human oncogene. *Nature*, **310**, 469.

STACEY, D.W. & KUNG, H.F. (1984). Transformation of NIH3T3 cells by microinjection of Ha-*ras* p21 protein. *Nature*, **310**, 508.

SUKUMAR, S., NOTARIO, V., MARTIN-ZANCA, D. & BARBACID, M. (1983). Induction of mammary carcinomas in rats by nitrosomethylurea involves malignant activation of H-ras-1 loci by single point mutations. *Nature*, **306**, 658.

SWEET, R.W., YOKOYAMA, S., KAMATA, R., FERAMISCO, J.R., ROSENBERG, M. & GROSS, M. (1984). The product of *ras* is a GTPase and the T24 oncogene mutant is deficient in this activity. *Nature*, **311**, 273.

TABIN, C.J., BRADLEY, S., BARGMAN, C. & 6 others. (1982). Mechanisms of activation of human oncogene. *Nature*, **300**, 143.

TANAKA, T., SLAMON, D.J., BATTIFORA, H. & CLINE, M.J. (1986). Expression of p21 *ras* oncoproteins in human cancers. *Cancer Res.*, **46**, 1465.

TAPAROWSKY, E., SUARD, Y., FUSANO, O., SIMIZU, K., GOLDFARB, M. & WIGLER, M. (1982). Activation of the T24 bladder carcinoma transforming gene is linked to a single amino acid change. *Nature*, **300**, 762.

THOR, A., HORAN-HAND, P., WUNDERLICH, D., CARUSO, A., MURARO, R. & SCHLOM, J. (1984). Monoclonal antibodies define differential *ras* expression in malignant and benign colonic diseases. *Nature*, **311**, 562.

THORGEIRSSON, U.P., TURPEENNIEMI-HUJANEN, T., WILLIAMS, J.E. & 4 others. (1985). NIH/3T3 cells transfected with human tumor DNA containing activated *ras* oncogenes express the metastatic phenotype in nude mice. *Mol. Cell Biol.*, **5**, 259.

VIOLA, M.V., FROMOWITZ, F., ORAVEZ, S., DEB, S. & SCHLOM, J. (1985). Ras oncogene p21 expression is increased in premalignant lesions and high grade bladder carcinoma. *J. exp. Med.*, **161**, 1213.

VIOLA, M.V., FROMOWITZ, F., ORAVEZ, S. & 6 others. (1986). Expression of *ras* oncogene p21 in prostatic cancer. *New Engl. J. Med.*, **314**, 133.

WEINBERG, R.A. (1985). The action of oncogenes in the cytoplasm and nucleus. *Science*, **230**, 770.

WILLIAMS, A.R.W., PIRIS, J., SPANDIDOS, D.A. & WYLLIE, A.H. (1985). Immunohistochemical detection of the *ras* oncogene p21 product in an experimental tumour and in human colorectal neoplasms. *Br. J. Cancer*, **52**, 687.

WILLINGHAM, M.C., PASTAN, I., SHIH, T.Y. & SCOLNICK, E.M. (1980). Localisation of the *src* gene product of the Harvey strain of MSV to plasma membrane of transformed cells by electron microscopic immunocytochemistry. *Cell*, **19**, 1005.

WILLINGHAM, M.C., BANKS-SCHLEGEL, S.P. & PASTAN, I.H. (1983). Immunocytochemical localization in normal and transformed human cells in tissue culture using a monoclonal antibody to the *src* protein of the Harvey strain of murine sarcoma virus. *Exp. Cell Res.*, **149**, 141.

YUSPA, S.H., KILKENNY, A.E., STANLEY, J. & LICHTI, U. (1985). Keratinocytes blocked in phorbol ester-responsive early stage of terminal differentiation by sarcoma viruses. *Nature*, **314**, 459.